# Gas-Sensing Properties and Mechanisms of 3D Networks Composed of ZnO Tetrapod Micro-Nano Structures at Room Temperature

**DOI:** 10.3390/ma17010203

**Published:** 2023-12-30

**Authors:** Jinjiang Hu, Hong Ma, Yang Zhou, Liyong Ma, Shuyin Zhao, Shuzheng Shi, Jirong Li, Yongqin Chang

**Affiliations:** 1 School of Materials Science and Engineering, University of Science and Technology Beijing, Beijing 100083, China; 2 Department of Mathematics and Physics, Hebei University of Architecture, Zhangjiakou 075000, China; mahong@163.com (H.M.); zysun19940504@163.com (Y.Z.); maliyong@buaa.edu.cn (L.M.); zsy312@126.com (S.Z.); shishuzheng2000@163.com (S.S.); 15530319142@163.com (J.L.); 3 Zhangjiakou Smart Control Technology Innovation Center, Zhangjiakou 075000, China

**Keywords:** gas sensor, ZnO tetrapod, 3D networks, micro-nano structures, gas-sensing mechanisms, room temperature

## Abstract

Metal oxide semiconductors (MOSs) hold great promise for electronic devices such as gas sensors. The utilization of ZnO as a conductometric gas sensor material can be traced back to its early stages; however, its application has primarily been limited to high-temperature environments. A gas sensor based on highly porous and interconnected 3D networks of ZnO tetrapod (ZnO-T) micro-nano structures was fabricated via an easy chemical vapor deposition (CVD) method. Homemade instruments were utilized to evaluate the gas-sensing of the sample at room temperature. It exhibited good gas-sensing at room temperature, particularly with a response of up to 338.80% toward 1600 ppm ethanol, while also demonstrating remarkable repeatability, stability, and selectivity. Moreover, the unique gas-sensing properties of ZnO-T at room temperature can be reasonably explained by considering the effect of van der Waals forces in physical adsorption and the synergistic effect of carrier concentration and mobility. The aforementioned statement presents an opportunity for the advancement of gas sensors utilizing ZnO at room temperature.

## 1. Introduction

Air pollution, which has a serious impact on both ecosystems and human health, is increasingly becoming a pressing global issue alongside water and soil pollution [1,2,3]. Furthermore, gas leaks and explosions are also prevalent in both production and daily life [4,5,6]. For the sake of environmental protection, production safety, and public health, it is crucial to develop highly sensitive methods for detecting toxic, harmful, flammable, and explosive gases or volatile organic compounds (VOCs) [7,8,9]. Gas sensors that detect and measure gases or VOCs are essential for environmental monitoring [10], industrial production [11], safety control [12], biotechnology [13], healthcare [14], and other fields.

Due to their exceptional electrical properties, cost-effectiveness, smooth operation, and scalability, MOSs are currently the most widely used materials for fabricating resistivity-based gas sensors [15,16,17]. Moreover, MOS sensors, renowned for their simple structure, low power consumption, and heightened sensitivity, have made significant progress in the rapid and convenient detection of VOCs, which are ubiquitous in various aspects of human existence. Once their concentration exceeds a certain threshold, they can pose severe health hazards and even endanger lives [18,19,20]. As one of the most critical n-type semiconductors with high electron mobility, ZnO has been widely utilized in gas-sensing applications since the 1960s [21]. In recent decades, with the advancement of nanoscience and nanotechnology, a diverse range of nano-ZnO gas sensors have been fabricated, including nanowires [22], nanorods [23], nanotubes [24], nanosheets [25], nanoflowers [26], and so on. Typically, these sensors function within the temperature range of 150–500 °C or can be operated at room temperature with auxiliary means such as UV light irradiation [27,28,29]. However, this approach may lead to increased complexity in fabrication and power consumption, and reduced sensor stability and lifespan, ultimately limiting their widespread applications. By lowering the operational temperature of the sensing material, it becomes feasible to simplify, miniaturize, and reduce the power consumption of the device in comparison with auxiliary methods. Consequently, there has been a significant surge in demand for gas sensors that can operate at room temperature without any adjuncts.

Various approaches have been identified for reducing the operating temperature of nano-ZnO gas sensors to room temperature, including metal modification, material composites, and photoactivation [30,31,32]. However, these methods increase manufacturing complexity and costs. Fortunately, due to its unique structure and exceptional performance, ZnO-T has garnered significant interest from users and scientists alike. It is widely utilized in various fields such as insulation materials [33], medical treatments [34], photocatalysis [35], photoluminescence [36], and gas detection [37]. In fact, it can be considered the most important structure from an application perspective [38]. In this study, ZnO-T micro-nano structures were synthesized via a facile CVD process, and their gas-sensing properties toward ethanol, methanol, and oxygen at room temperature were investigated. Moreover, prior research has predominantly investigated the gas-sensing mechanism through an analysis of carrier concentrations and a focus on alterations in such concentrations resulting from chemisorption [39]. However, at room temperature, mobility has emerged as an increasingly crucial factor and thus demands greater attention for physical adsorption. By considering both chemical and physical adsorption, as well as the synergistic effects of carrier concentration and mobility, this study emphasizes the room-temperature gas-sensing mechanisms of ZnO-T micro-nano structures. The conclusion drawn is consistent with the experimental data.

## 2. Experimental Section

### 2.1. Synthesis Process

**Figure 1:**
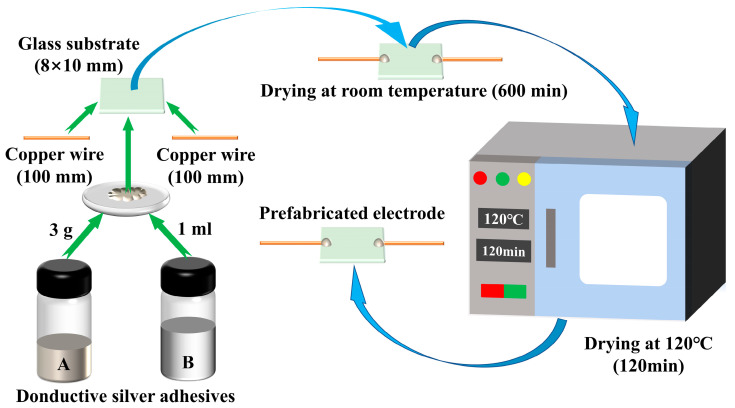
Schematic diagram of electrode preparation process.

**Figure 2:**
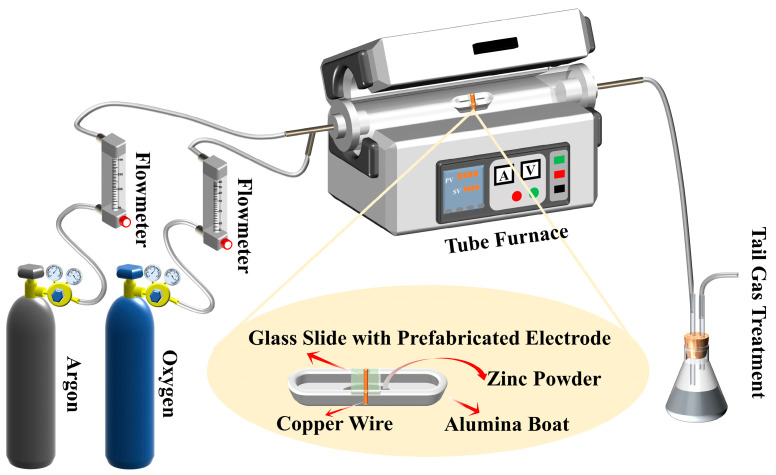
Schematic diagram of CVD.

A glass substrate was coated with a thin film of ZnO-T micro-nano structures, featuring highly porous and interconnected 3D networks, via CVD deposition. Details on the preparation of the electrodes used in this process are shown in Figure 1. The silver powder conductive adhesive (DB5015, Wuhan Double Bond Chem Sealing Material Co., LTD., Wuhan, China) was thoroughly mixed in a ratio of A:B=3.5 g:1 mL. Subsequently, the glass sheet (8×10 mm) and two copper wires were bonded together using this mixture. The assembly was then air-dried for 600 min at room temperature before being transferred to an electric blast drying oven (T-1, Dongguan Haobang Instrument and Equipment Co., LTD., Dongguan, China). After drying at 120 °C Celsius for 120 min, the prefabricated electrode was obtained following natural cooling. As shown in Figure 2, Zn powders (AR, Sinopharm Chemical Reagent Co., LTD, Shanghai, China) were loaded into an alumina boat and covered with a glass slide, serving as the source material. The boat was then placed at the center of a quartz tube mounted on the tube furnace (GSL-1300X, Hefei Kejing Material Technology Co., LTD, Hefei, China). During the synthesis process, a continuous flow of argon (99.999%, Beijing Huanyu Jinghui capital gas Technology Co., LTD, Beijing, China) at 100 sccm was vented in the quartz tube. The furnace was heated at a rate of 13 °C/min until it reached 640 °C and this temperature was maintained for 15 min. Oxygen (99.999%, Beijing Huanyu Jinghui capital gas Technology Co., LTD, Beijing, China) with a flow rate of 8 sccm was introduced into the quartz tube during the growth process. Once completed, the boat was immediately removed and cooled to room temperature in air.

### 2.2. Characterization

The as-synthesized sample was analyzed through X-ray diffraction (XRD, Rigaku, D/MAX-RB, Tokyo, Japan) using Cu-Kα radiation with a scan range of 20° to 70° and a scanning rate of 0.02°, elucidating its crystalline structure. The surface morphology and elemental composition of the sample were analyzed using a field emission scanning electron microscope (FESEM, JSM-7001F, JEOL, Tokyo, Japan) equipped with an energy dispersive spectroscopy (EDS) instrument. The morphologies and sizes of the as-obtained sample were observed using transmission electron microscopy (TEM; JEM-2100F, JEOL, Tokyo, Japan) at 200 kV to obtain high-resolution images. FTIR absorption spectra of the sample were obtained using a Fourier infrared spectrometer (Thermo Scientific Nicolet iS20, Thermo Fisher Scientific, Waltham, MA, USA). Photoluminescence (PL) measurements were conducted on the sample using a fluorescence spectrometer (Hitachi F-4500, Hitachi, Tokyo, Japan) equipped with a xenon lamp as the excitation source.

### 2.3. Gas-Sensing Measurements

**Figure 3:**
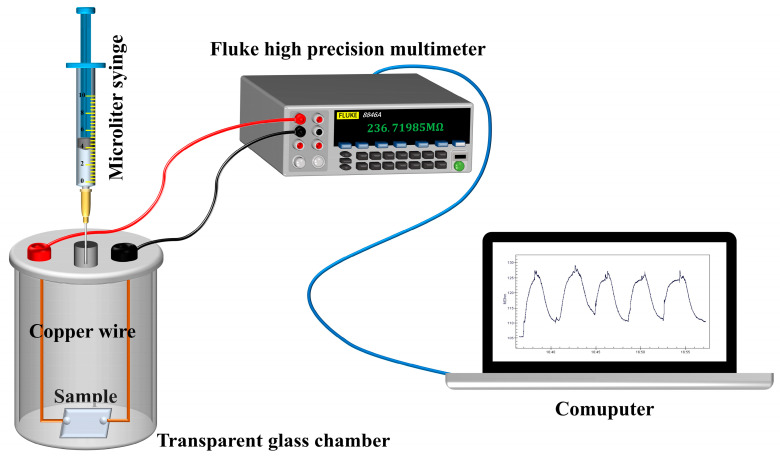
Schematic diagram of static chamber gas testing system.

**Figure 4:**
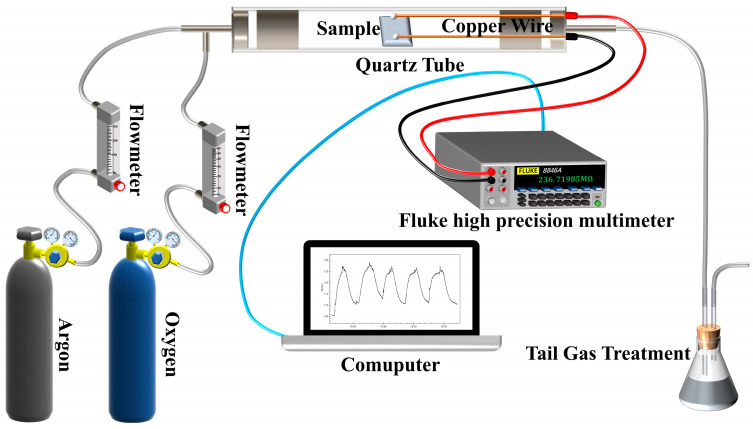
Schematic diagram of dynamic gas testing system.

The gas-sensing of the sample was evaluated using two custom-built instruments, one designed for detecting VOCs (AR, Sinopharm Chemical Reagent Co., LTD, Shanghai, China), while the other was used to measure oxygen, as illustrated in Figure 3 and Figure 4, respectively. The resistance measuring equipment of both measurement systems employs the Fluke Multimeter (8846A, Fluke Electronic Instrumentation, Evered, WA, USA) The performance tests were conducted at ambient temperature, specifically set to 293 K, with a relative humidity of 23%, unless otherwise specified.

The test chamber volume for static gas in Figure 3 was characterized as follows:

(1) $$V_{\mathrm{liquid},\mathrm{eth}}=1.19\frac{C_{\mathrm{eth}}}{T}$$

(2) $$V_{\mathrm{liquid},\mathrm{met}}=0.83\frac{C_{\mathrm{met}}}{T}$$

(3) $$V_{\mathrm{liquid},\mathrm{ace}}=1.50\frac{C_{\mathrm{ace}}}{T}$$

(4) $$V_{\mathrm{liquid},\mathrm{ben}}=1.81\frac{C_{\mathrm{ben}}}{T}$$

(5) $$V_{\mathrm{liquid},\mathrm{tol}}=2.16\frac{C_{\mathrm{tol}}}{T}$$

where *V*_liquid_ represents the volume of ethanol or methanol in the liquid state, and *C* denotes the concentration of ethanol or methanol in the gaseous state.

The gas flow rate of the dynamic multiple gas distributing scheme depicted in Figure 4 was measured via a mass flowmeter. Specifically, the argon flow rate was pre-set to 200 sccm while the oxygen flow rates were set at 15 sccm, 30 sccm, 45 sccm, and 60 sccm.

The $R_{g}$ is defined as the resistance of the sensor when exposed to the tested gas, while the $R_{a0}$ and $R_{at}$ represent the resistance values before target gas injection and after pumping, respectively. In this manner, the response and recovery times are defined as the duration required for the sensor’s resistance to attain 90% of its total altered value, ($R_{g}-R_{a0}$) and ($R_{g}-R_{at}$), respectively. The responsivity is expressed as *S* (%) using the following formula:

(6) $$S=\frac{R_{g}}{R_{a0}}\times 100\%$$

## 3. Results and Discussion

### 3.1. Morphology and Structure of the Thin Film

**Figure 5:**
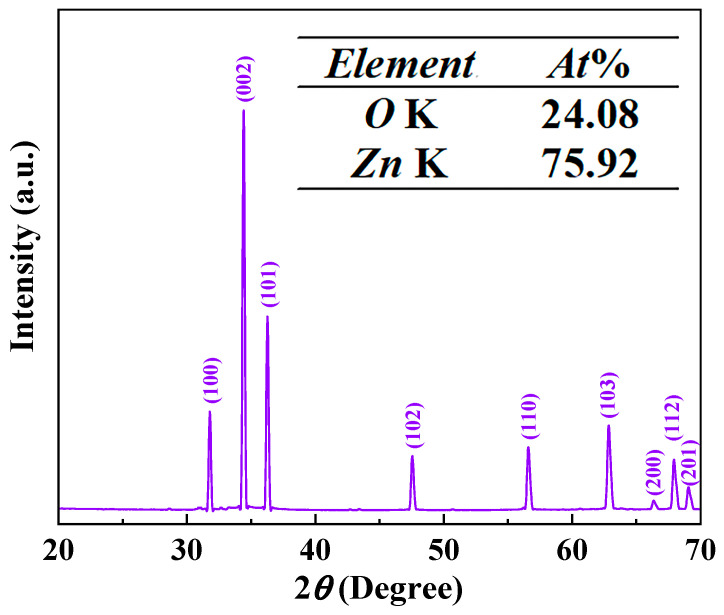
XRD patterns of the sample. Inset is the EDS spectrum results.

The purity and crystal structure of the as-grown ZnO film were confirmed using its XRD patterns. According to Figure 5, the primary diffraction peaks located at 31.8°, 34.5°, 36.3°, 47.6°, 56.6°, 62.9°, 66.4°, 67.9°, and 69.1° correspond to the (100), (002), (101), (102), (110), (103), (200), (112), and (201) planes of the standard XRD patterns of the high-crystallinity wurtzite ZnO (JCPDS card #36-1451). The inset is the outcome of the EDS analysis, revealing that the film solely consists of Zn and O elements. As evidenced by the XRD and EDS results, CVD-synthesized ZnO exhibits a high purity and crystallinity without any impurities. However, the stoichiometric ratio deviates significantly from the expected values for ZnO, suggesting an initial abundance of zinc vapor but insufficient introduction of oxygen into the furnace. Consequently, only a limited number of gaseous ZnO molecules were formed.

**Figure 6:**
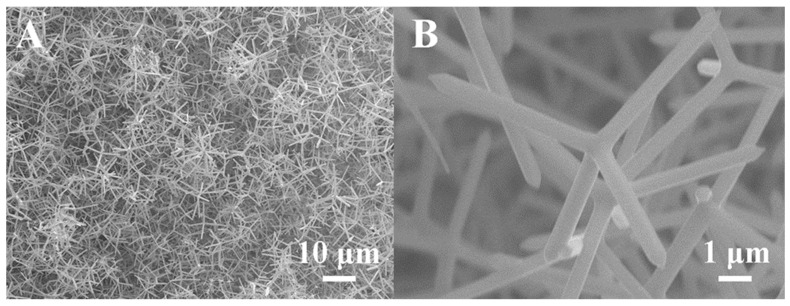
SEM images of the as-prepared sample, 3D networks of ZnO-T micro-nano structures in (**A**) and single ZnO-T in (**B**).

**Figure 7:**
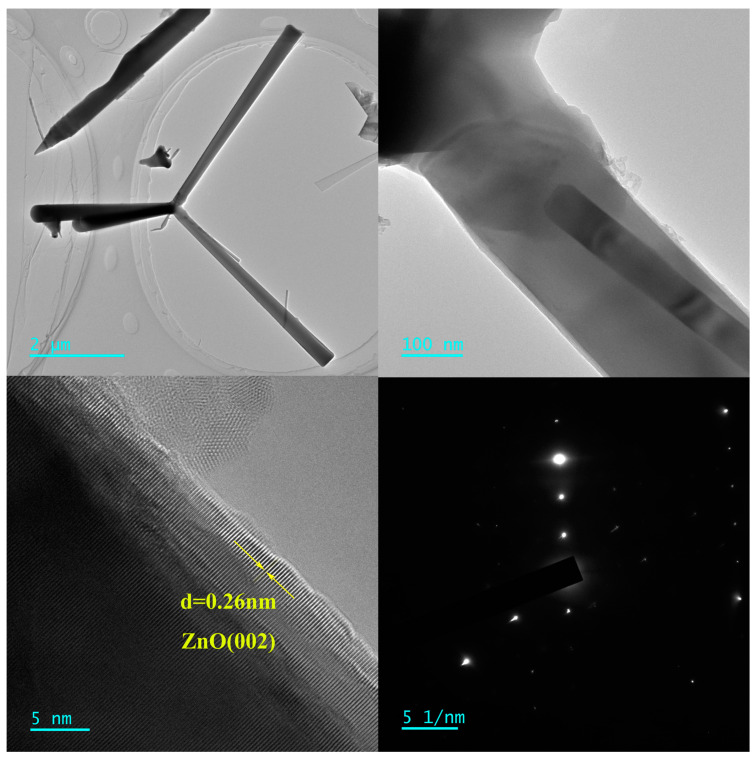
TEM images of the as-prepared sample.

Figure 6 presents SEM images of the film at varying magnifications, revealing highly porous and interconnected 3D networks of ZnO-T micro-nano structures in A, which contribute to its high surface-to-volume ratio and gas adsorption capacity. The tetrapod exhibited a fundamentally similar morphology, with arms of a uniform diameter tapering to a point at the distal end. Among the limited number of tetrapodal structures, nanowires grew from the arms with significantly reduced diameters and varying lengths. An example of a tetrapod structure is presented in B, featuring arms measuring approximately 560 nm in diameter and 5.4 μm in length, with an angle of roughly 109° between them. Figure 7 gives more detailed microstructural information presented using HRTEM. It can be seen that the lattice fringes of ZnO nanoparticles exhibit a d-spacing of 0.26 nm, which is indexed to the (002) crystal planes of ZnO. It is obvious that there was no streaking in the nanorod, which indicated a low density of structural defects in it, such as stacking faults and dislocations, and the T-ZnO nanorods were high-quality nanocrystals.

### 3.2. FTIR Analysis

**Figure 8:**
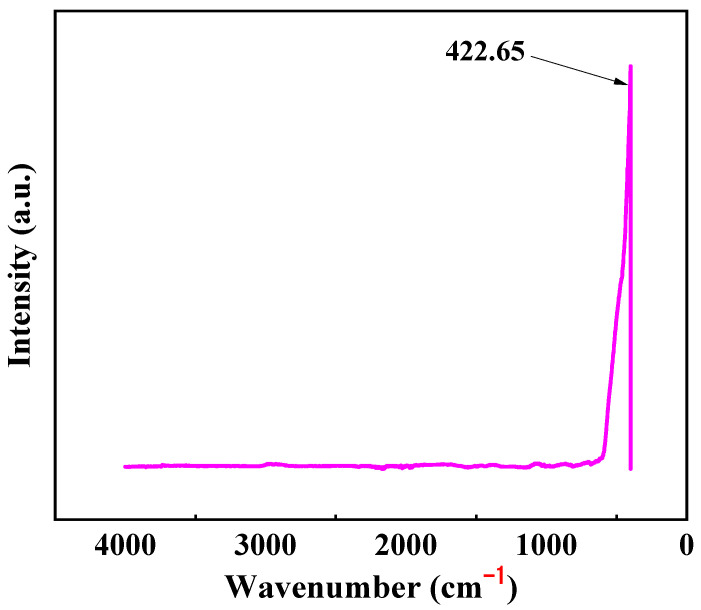
FTIR spectra of the ZnO film.

The infrared spectrum of ZnO thin films is depicted in Figure 8, wherein the prominent absorption peak at 422.65 cm^−1^ corresponds to the characteristic Zn-O bond. Comparatively, other peaks are scarcely discernible, indicating the high purity of the sample with no formation of additional bonds.

### 3.3. PL Analysis

**Figure 9:**
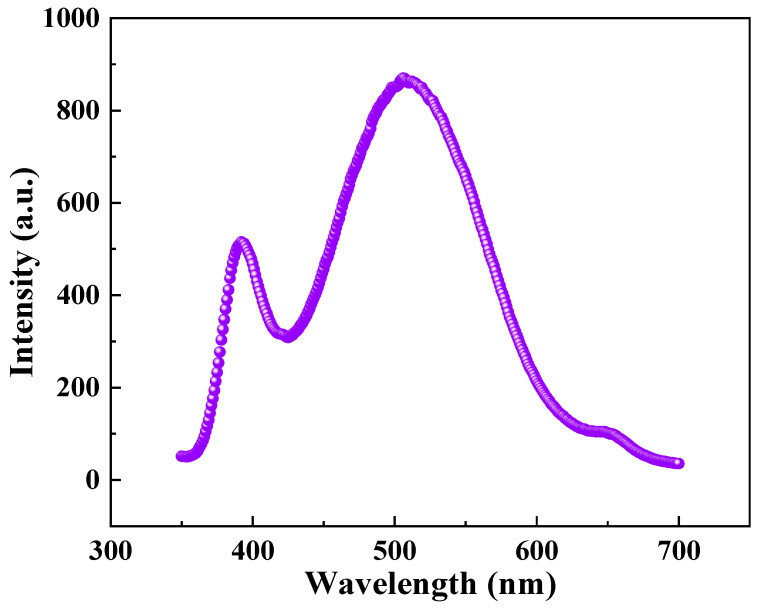
PL spectra of the ZnO film.

The quality of materials can be evaluated using their PL properties. As illustrated in Figure 9, the PL spectrum of the sample exhibited UV and visible (green) emission bands. The UV emission peak of ZnO is commonly attributed to excitons in close proximity to the band edge, while the green emission peak arises from a variety of defects present within the sample (such as oxygen vacancies, zinc interstitials, and doping-induced defects) [40]. To determine the degree of defects in ZnO materials, the intensity ratio between the band-edge emission and deep-level emission is commonly utilized, with a recorded value of 1.69 for this particular sample. The presence of a significant number of defects, such as oxygen vacancies and interstitial zinc, in the film is indicative of its high activity and sensitivity to visible light. These defects typically act as electron donors in ZnO, which may account for the favorable response of ZnO films toward ethanol’s physical adsorption at the ambient temperature.

### 3.4. Gas-Sensing Properties

**Figure 10:**
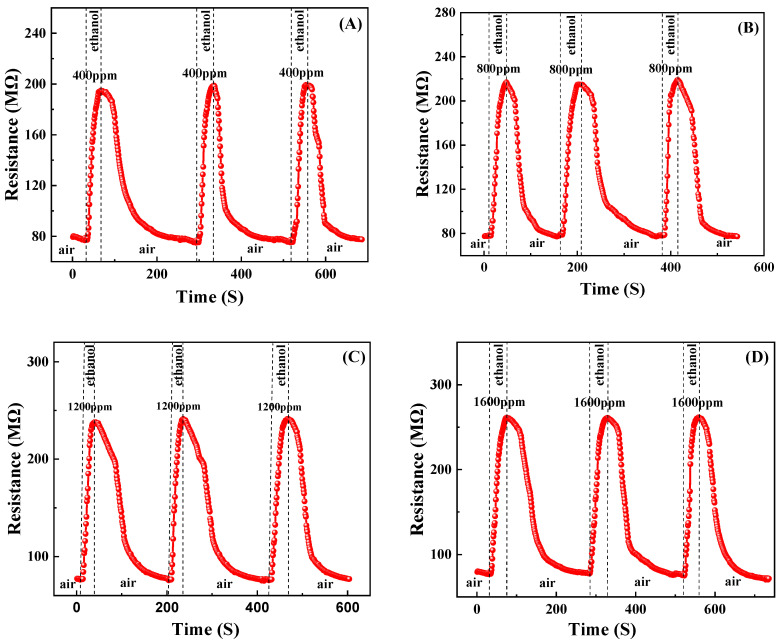
Reversible cycles for ethanol: (**A**) 400 ppm, (**B**) 800 ppm, (**C**) 1200 ppm, (**D**) 1600 ppm.

**Figure 11:**
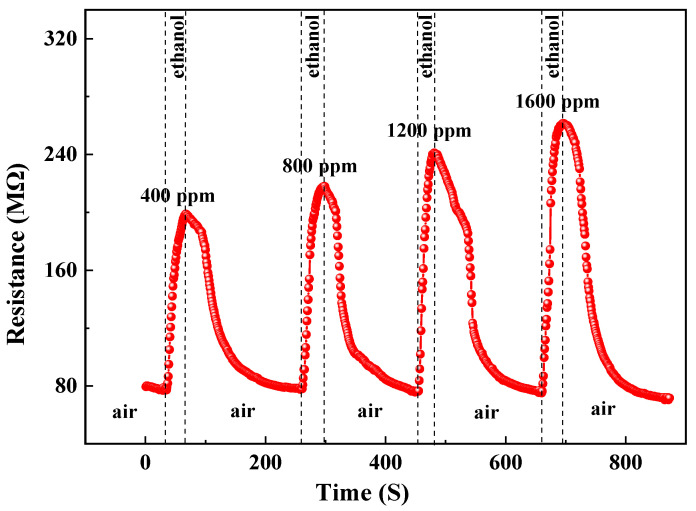
Response curves of the sample to ethanol.

**Figure 12:**
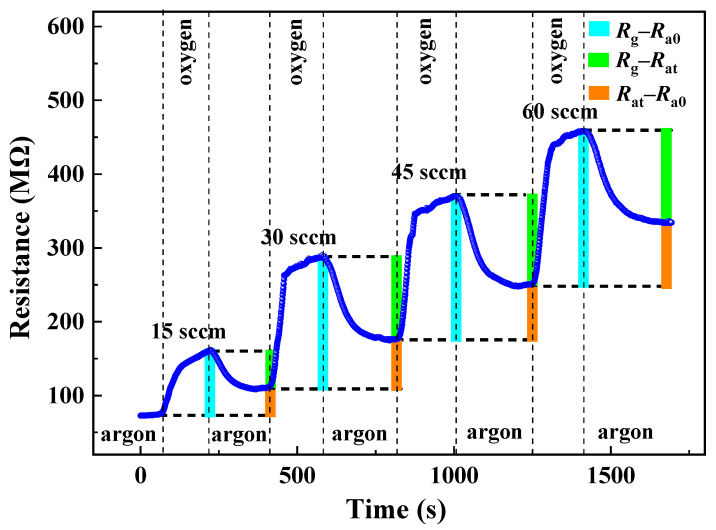
Response curves of the sample to oxygen.

The reversible cycles for ethanol are illustrated in Figure 10. It can be observed that the gas-sensing response of the sample exhibited stability and repeatability. Figure 11 and Figure 12 depict the resistance response curves of the sensor under varying concentrations of ethanol (400 ppm, 800 ppm, 1200 ppm, and 1600 ppm) and oxygen flow rates (15 sccm, 30 sccm, 45 sccm, and 60 sccm), respectively. When the sample was exposed to the target gas, whether it is ethanol or oxygen, a noticeable increase in resistance occurred. This finding is consistent with previous studies on oxygen but contradicts those on ethanol at high temperatures where the resistance decreases [41,42]. Furthermore, the adsorption and desorption of the film for ethanol exhibited reversibility ($R_{at}=R_{a0})$, while its total resistance remained almost constant. However, it showed irreversibility for oxygen ($R_{at}>R_{a0})$ with a continuous increase in resistance.

Table 1 presents the response and recovery times of the sample to ethanol, methanol, and oxygen. The response time to different concentrations of ethanol was remarkably shorter, approximately 20 s, which is less than half that of oxygen. However, both ethanol and oxygen exhibited a prolonged recovery time exceeding one minute. In general, the response and recovery time to ethanol was superior to that of oxygen.

**Figure 13:**
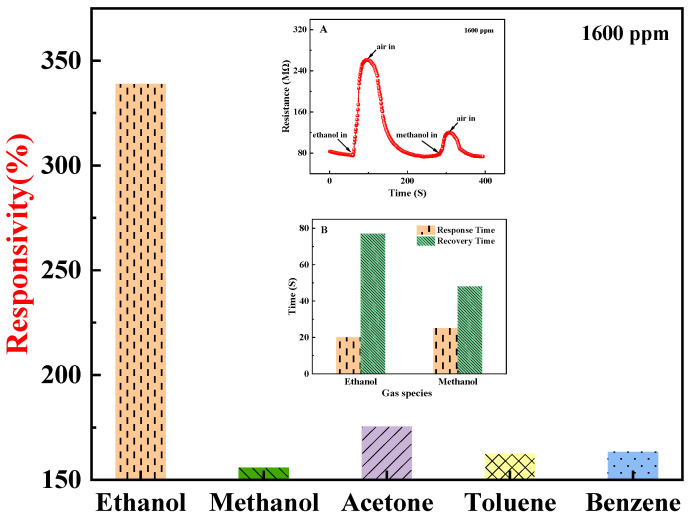
Selectivity test of the sample toward different VOCs with 1600 ppm concentration. Inset is a comparison of sample responsiveness (**A**) and response recovery times for ethanol and methanol (**B**).

The ZnO-T-based sensor was exposed to different vapors, like ethanol, methanol, acetone, toluene, and benzene, of 1600 ppm concentration at room temperature and results are shown in Figure 13. Interestingly, the sample shows high selectivity toward ethanol as compared to other analytes. The inset is a comparison of the sample responsiveness (A) and response recovery times for ethanol and methanol (B), which shows that the recovery time for methanol was significantly shorter.

**Figure 14:**
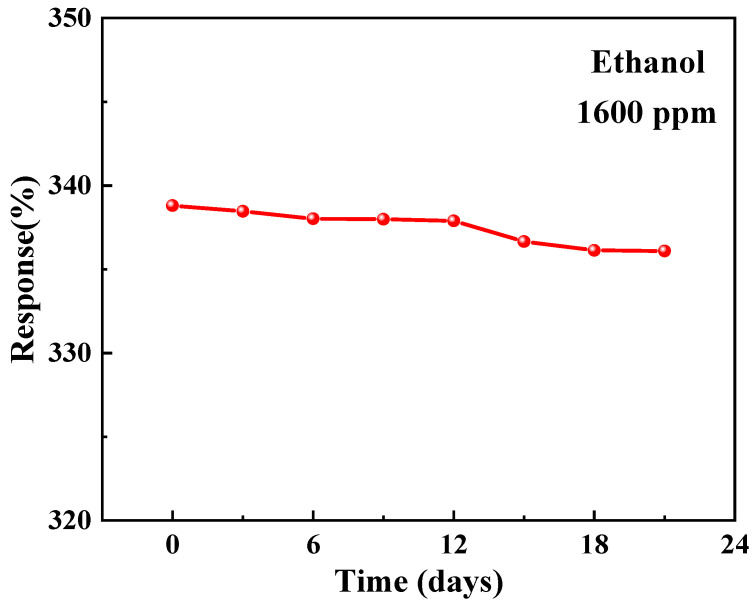
Stability performance of the sensor during a testing period of 21 days.

**Figure 15:**
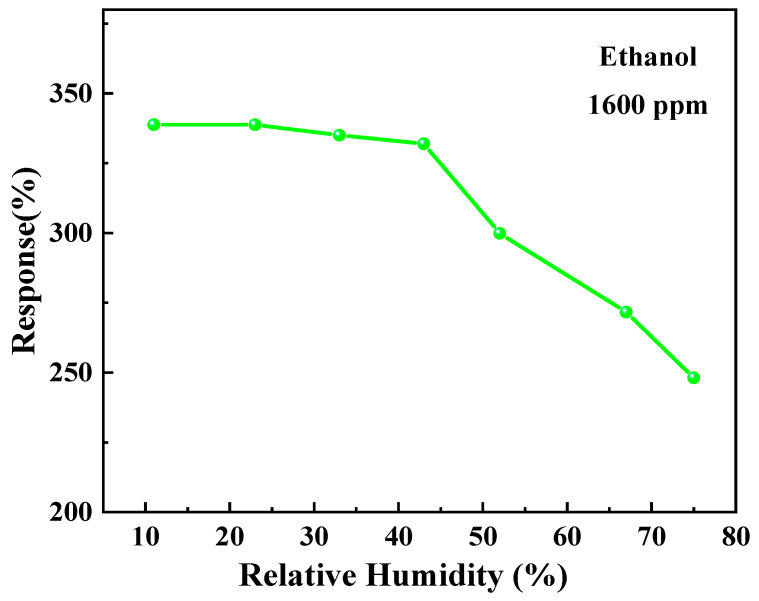
Ethanol response properties of the sensor in different humidity conditions.

The stability of the sensor to ethanol over a period of 21 days is illustrated in Figure 14. It can be observed that the responsivity to ethanol gradually declined after day 12, eventually reaching approximately 95% of the initial value by day 21. The response of the sample to ethanol under different humidity conditions is illustrated in Figure 15. The responsivity significantly decreased when the relative humidity exceeded 52%, and further dropped to approximately 73% of the original responsivity at a humidity level of 75%. The gas-sensing properties of ZnO-T-based sensors are compared to those of previously reported ZnO nanostructures for ethanol in Table 2 [43,44]. It is evident that ZnO-T exhibits a superior response toward ethanol and operates at room temperature.

### 3.5. Gas-Sensing Mechanisms

According to previous research, an electron depletion layer (EDL) forms on the surfaces of n-type MOS in air due to the abstraction of electrons by adsorbed oxygen species on the gas sensor surface. When exposed to reducing gases, the reaction between the gas and adsorbed oxygen species releases electrons that return to the sensor surface, resulting in a reduction in the EDL width and a decrease in sensor resistance. Conversely, exposure to oxidizing gases increases the EDL width and leads to an increase in sensor resistance [22,23]. However, in our study, the resistance of the sample increased upon exposure to ethanol or oxygen. The adsorption/desorption process with ethanol was reversible and maintained a nearly constant total resistance. In contrast, the adsorption/desorption process with oxygen was irreversible and resulted in a continuous increase in total resistance.

The aforementioned inconsistency can be attributed to the distinct gas-sensing mechanisms of gas sensors that function at varying temperatures and with different adsorption types, including chemisorption or physical adsorption. It is widely acknowledged that the electrical conductivity of an n-type semiconductor can be mathematically expressed as follows [40]:

(7) $$\sigma =\frac{ne^{2}\tau }{m^{*}}$$

where *n* represents the electron number density, *e* denotes the electron charge, τ stands for the relaxation time, and $m^{*}$ refers to the electron effective mass. Both *n* and τ are two decisive factors that significantly impact material conductivity.

In terms of gas-sensing, the sorption of oxygen plays a pivotal role in determining the electrical transport properties of ZnO nanostructures. Below 150 °C, the $O_{2}^{-}$ molecular species dominates while above this temperature, the atomic $O^{-}$ species takes over. The reaction kinetics are as follows [39]:

(8) $$O_{2(\mathrm{gas})}⇄O_{2(\mathrm{ads}.)}$$

(9) $$O_{2(\mathrm{ads}.)}+e^{-}⇄O_{2}^{-}(O_{2}^{-}\mathrm{dominate}\;\mathrm{at}\;25–150\;℃)$$

(10) $$O_{2}^{-}+e^{-}⇄2O^{-}(O^{-}\mathrm{dominate}\;\mathrm{at}\;150–500\;℃)$$

The adsorption of the target gas on MOS gas sensors typically takes two forms: physical and chemical. At temperatures above 150℃, exposure to ethanol triggers redox reactions between ethanol molecules and adsorbed oxygen atoms, $O^{-}$ (as shown in Equation (11)) [39]. Ethanol molecules undergo chemical adsorption, resulting in the release of electrons that return to the sensor surface. This leads to an increase in electron number density (*n*) and a subsequent reduction in sensor resistance. In contrast, exposure of the sample to oxygen leads to a continuation of the reaction represented in Equation (10), with increasing oxygen concentration resulting in sequential chemisorption of oxygen molecules and a decrease in electron number density (*n*), ultimately leading to an increase in sensor resistance. The redox reaction exhibits high activity at elevated temperatures, and the chemisorption of the target gas on ZnO films confers an absolute advantage that far surpasses physical adsorption. The electron number density (*n*) serves as a decisive factor in determining sensor resistivity under high-temperature conditions.

(11) $${C_{2}H_{5}\mathrm{OH}}_{\left(\mathrm{gas}\right)}+6O^{-}⇄3{\mathrm{CO}}_{2(\mathrm{gas})}+3H_{2}O_{\left(\mathrm{gas}\right)}+6e^{-}$$

**Figure 16:**
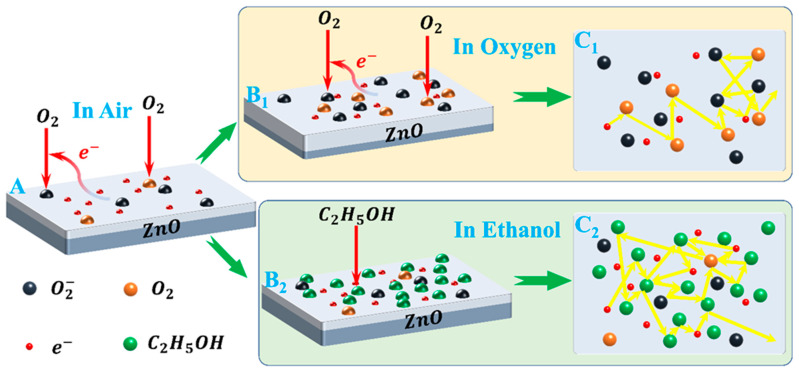
Gas-sensing mechanism diagram of ZnO film to ethanol and oxygen at room temperature.

**Figure 17:**
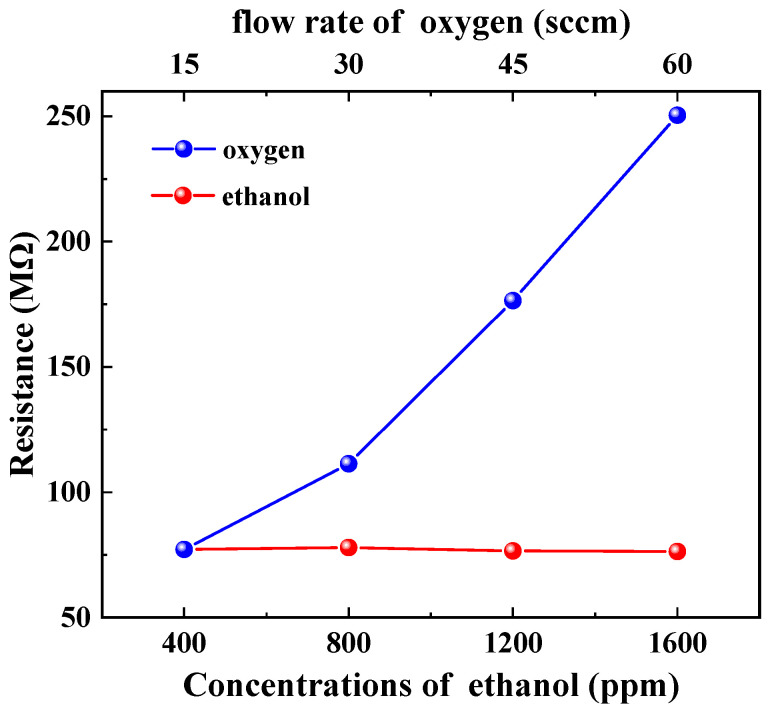
Resistance characteristics of ZnO film to ethanol and oxygen.

However, at room temperature, the redox reactions between ethanol molecules and adsorbed molecular $O_{2}^{-}$ exhibit relatively weak interactions. The primary adhesion force between ethanol molecules and ZnO particles on the film surface or adsorbed molecular $O_{2}^{-}$ is van der Waals forces. Therefore, physical adsorption is the predominant form of ethanol molecule adsorption on ZnO films, surpassing chemical adsorption by a significant margin. The variation in electron number density (*n*) may be neglected, while the alteration in conductivity is primarily dependent on the electronic relaxation time (*τ*) (refer to Figure 16, $A\to B_{2}\to C_{2}$). According to the mode of free electrons with random point scattering [40], the adsorption of ethanol molecules on the surface of thin films increases electron scattering centers, thereby reducing electron relaxation time (*τ*) and resulting in an increase in sample resistivity (refer to Figure 11). Moreover, the increase in ethanol concentration leads to enhanced adsorption, an increased number of scattering centers, and higher resistivity. This provides a plausible explanation for the observed phenomenon of increased sample resistance in an ethanol atmosphere, with changes in resistance being directly proportional to ethanol concentration (refer to Figure 17).

On the other hand, the oxygen adsorption process on ZnO thin films primarily involves two mechanisms: $O_{2(\mathrm{ads}.)}$ accepting electrons from the thin film to generate $O_{2}^{-}$ (as shown in Equation (9)), and $O_{2(\mathrm{ads}.)}$ being attracted by van der Waals forces to the surface of the film or pre-existing $O_{2}^{-}$. Both processes lead to an increase in sample resistance, which is positively correlated with the oxygen flow rate (refer to Figure 12). These two processes correspond to chemisorption and physisorption, respectively. They decrease the electron number density (*n*) and electronic relaxation time (*τ*), thereby increasing the sample’s resistance (refer to Figure 16, $A\to B_{1}\to C_{1}$). The resistance variation caused by physical adsorption can be restored during the oxygen pumping stage, whereas that induced by chemical adsorption cannot be reverted to its initial value. This is also why the total resistance of the sample remained constant during the ethanol measurement and positively correlated with the oxygen flow rate during the oxygen measurement (refer to Figure 17).

**Figure 18:**
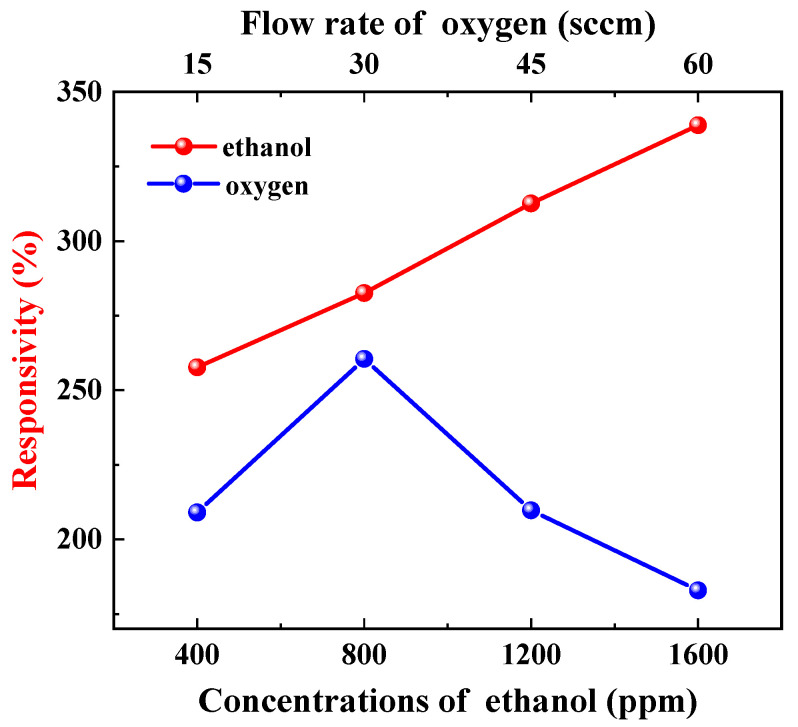
Response of ZnO film to ethanol and oxygen.

**Figure 19:**
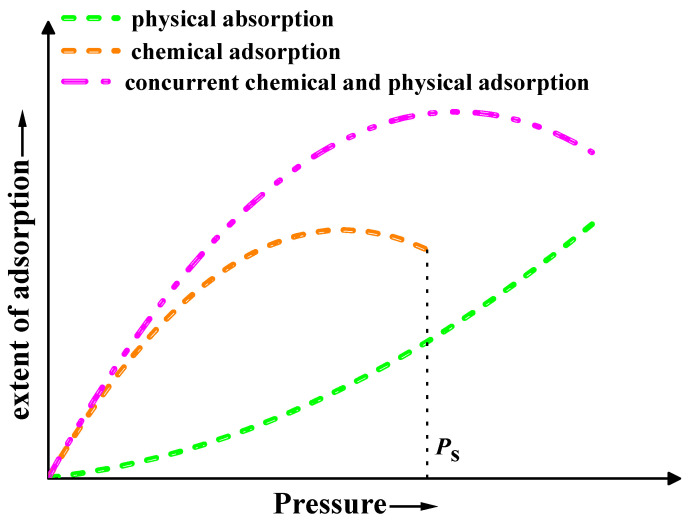
Extent of adsorption variation vs. pressure.

**Figure 20:**
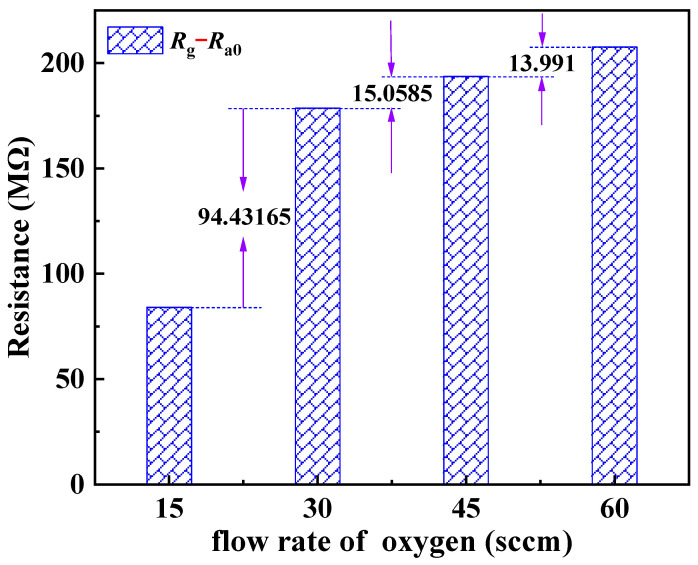
Resistance variation of ZnO film toward oxygen.

Two additional phenomena support the aforementioned adsorption mode determination. Firstly, there were distinct variations in the sensor’s responsivity to ethanol and oxygen. The sensor’s responsivity to ethanol increased with rising concentrations, reaching a maximum of 338.80% at 1600 ppm. Nevertheless, its responsivity to oxygen initially rose with an increasing flow rate before peaking at 260.45% at 30 sccm and subsequently declining (refer to Figure 18). The increase in sample resistance caused by physical adsorption of ethanol was concentration-dependent and reversible. Consequently, the responsivity also increased with increasing ethanol concentration, which is consistent with the relationship between the extent of adsorption and pressure in physical adsorption (as shown by the green line in Figure 19) [45]. The change in resistance of the sample, resulting from the synergistic effect of oxygen chemisorption and physisorption, exhibited a non-linear growth rate with an increasing flow rate. The front section showed a greater increase than the back section (refer to Figure 20). Moreover, physical sorption was reversible whereas chemical sorption was irreversible, leading to a continuous increase in total resistance. The responsivity displayed a pattern of increase followed by a decrease, with the maximum value achieved at an oxygen flow rate of 30 sccm. This phenomenon is in line with the correlation between adsorption extent and pressure during concurrent chemical and physical adsorption (as shown by the carmine line in Figure 19). Furthermore, the sensor exhibited nearly identical response times for ethanol and methanol, yet it demonstrated greater responsivity and a longer recovery time for ethanol (refer to illustrations A and B in Figure 13). This phenomenon serves as evidence of the physical adsorption of reducing gases by the sample. According to the characteristics of van der Waals forces, the intermolecular force is greater for molecules with a higher molecular weight or more polarity. Methanol molecules are non-polar while ethanol molecules are polar and have a higher molecular weight than methanol. Therefore, physical adsorption of ethanol on the sample is stronger than that of methanol, resulting in a higher response and longer recovery time for ethanol.

## 4. Conclusions

The highly porous and interconnected 3D networks of ZnO-T micro-nano structures were facilely fabricated via a CVD process. Pure ZnO exhibited good gas-sensing at room temperature, particularly with a response of up to 338.80% toward 1600 ppm ethanol, while also demonstrating remarkable repeatability, stability, and selectivity. This can be attributed to the unique tetrapod-shaped micro-nano structures. Moreover, through a comparison of the distinct responses of the sample to ethanol and oxygen, taking into account van der Waals forces in physical adsorption, and based on the synergistic effect of carrier concentration and mobility in various adsorption processes, we have investigated the gas-sensing mechanism of ZnO-T at room temperature. This provided a promising and cost-effective approach for detecting ethanol at room temperature.

## Figures and Tables

**Table 1:** Response and recovery time of the sample to ethanol, methanol, and oxygen.

	Concentration of Ethanol (ppm)				Flow Rate of Oxygen (sccm)			
	400	800	1200	1600	15	30	45	60
response time (S)	16	23	18	20	58	67	40	48
recovery time (S)	61	60	81	77	63	94	73	77

**Table 2:** Comparison of different ethanol gas sensors based on ZnO.

Materials	Working Temperature (K)	Ethanol (ppm)	Response	Reference
ZnO nanowire	333	500	0.75	[43]
ZnO-SPES	Room temperature	10,000	1.27	[44]
ZnO tetrapod	Room temperature	1600	3.38	This work

## Data Availability

Data are contained within the article.
